# Effects of Meridian Acupressure on Stress, Fatigue, Anxiety, and Self-Efficacy of Shiftwork Nurses in South Korea

**DOI:** 10.3390/ijerph18084199

**Published:** 2021-04-15

**Authors:** Youngmi Cho, Jung-Min Joo, Seyoon Kim, Sohyune Sok

**Affiliations:** 1 Department of Nursing, Sun Moon University, Chungcheongnam-do, Asan-si 31460, Korea; choyoung23@yahoo.com; 2 Department of Nursing, Graduate School, Kyung Hee University, Seoul 02447, Korea; joojungmin88@gmail.com (J.-M.J.); ssamyun85@naver.com (S.K.); 3 College of Nursing Science, Kyung Hee University, Seoul 02447, Korea

**Keywords:** acupressure, occupational stress, fatigue, anxiety, nurse

## Abstract

Shiftwork nurses experience physical and psychological health problems related to shift work. This study aimed to examine the effects of Meridian acupressure on stress, fatigue, anxiety, and self-efficacy of shiftwork nurses in South Korea. A quasi-experimental pretest-posttest control group design was employed. Study participants were a total of 59 shiftwork nurses (intervention group: *n* = 29, control group: *n* = 30) in S hospital, Seoul, South Korea. The study was conducted at nurse stations in S hospital. Meridian acupressure as intervention was conducted for a total of 15 min on six Meridian acupressure points (GV 20, GB 12, GB 21, LI 11, SI 3, KI 1), 2 min 30 s (10 times for 15 s at a time) on each Meridian point. Measures were the stress scale, fatigue scale, State Anxiety Inventory, and self-efficacy scale, in Korean. Data were collected from July to August 2018. There were significant differences in the degrees of stress, fatigue, and anxiety of shiftwork nurses between the two groups. Meridian acupressure significantly decreased stress, fatigue, and anxiety of shiftwork nurses. This study provides preliminary evidence that Meridian acupressure was an effective intervention. Meridian acupressure could be applied to shiftwork nurses in various clinical situations.

## 1. Introduction

Medical healthcare support provides a 24-h service. Most nurses work two to three shifts a day in order to constantly monitor the patient’s condition for 24 h and provide high-quality nursing [1,2]. Many people are cared for conveniently, with efficient utilization of time, through shift work implementation. However, the shift workers themselves experience physical and psychological health problems related to shift work by working in a time zone that does not match their body’s rhythm in daily life [3,4]. In addition, the health of nurses is being threatened due to the demand for new knowledge and skills, teamwork among different occupations, and excessive workload [1,3]. Various harmful environments, such as contact with patients and their caregivers at work and hospital-acquired infections, may cause mental or physical health problems [2,4]. Erratic work schedules in three shifts may result in the imbalance of physiological factors, sleep-wake cycle, and daily life rhythms, which can cause stress, fatigue, poor performance, and depression [4,5].

A certain degree of stress helps nurses in performing their tasks, and motivates them by increasing their growth and productivity as individuals [6]. However, if nurses are suffering from chronic stress, they will eventually burn themselves out, which may have a negative impact not only on the nurses themselves, but also on their work, including nursing services for patients and for the organization [5,6]. Increased stress can negatively affect the quality of life of nurses by reducing their physical and mental performance, and causing sustained fatigue [7,8]. This induced fatigue will lead to job dissatisfaction, thereby increasing burnout, absenteeism, and turnover rate [1,2,9]. Once fatigue starts to develop, it may cause an increase in various physical and mental discomforts, and interfere with work, exercise, and interpersonal relationships [5,8]. It may also cause maladaptation syndrome, including emotional disorders, digestive diseases, and cardiovascular diseases [1,2].

Anxiety is an emotional state that is subjectively experienced in response to increased stress. It refers to the ambiguous and vague emotions arising as a result of the loss of internal control rather than from external threats, such as concern, tension, worry, and fear of imminent danger [3,10,11]. Physical and psychological pathologies occur when the degree of anxiety is severe and adversely affect the healing and recovery of disease [3,12]. Therefore, early intervention is deemed necessary.

Self-efficacy is a belief that an individual can successfully perform the actions necessary to produce a result [13]. The higher the level of self-efficacy, the more likely it is that ways to cope with stress are diverse and positive, thereby showing higher problem-solving ability [14,15]. In other words, nurses with higher self-efficacy tend to have a more positive performance in their nursing career [14]. Enhanced self-efficacy leads to higher quality nursing and improves the performance of individuals and organizations, thereby reducing stress [14,16].

Studies on nursing interventions to reduce nurses’ stress, fatigue and anxiety and to improve self-efficacy, such as foot bath, foot mat, back massage with lavender oil, foot reflexology massage, ear massage with peppermint oil, and self-growth training programs have been applied as interventions that have shown positive effects [1,2,3,6,7]. The importance of stress and fatigue management in nurses is very common in these previous studies. For this reason, interventions that are not interrupted by time, place, or tools and do not have any side effects are necessary, as these will help nurses in managing stress easily and effectively in their busy schedule.

Acupressure speeds up metabolism, relaxes muscular rigidity, and controls the function of the internal organs [2,12,17]. It is an easy, simple, and effective intervention without any side effects, and does not require any special equipment [11,18]. Furthermore, it is easily applicable without physical damage. Acupuncture should be placed in the correct position but, in the case of finger pressure, pressing around the trigger points transmits the pressure and revives the active physiological function, even though these are not the exact spots [5,19]. Therefore, anyone can easily learn and try this.

Studies that applied acupressure as a nursing intervention showed that acupressure massage, self-acupressure, and finger-pressure therapy had positive effects on stress and immune responses, sleep disorders, mood, fatigue, and back pain [2,3,20,21,22]. However, there is a lack of research on shiftwork nurses. Therefore, this study was conducted in order to examine the effects of Meridian acupressure on shiftwork nurses. The aims were to examine the effects of Meridian acupressure on stress, fatigue, anxiety, and self-efficacy in shiftwork nurses.

## 2. Material and Methods

### 2.1. Study Design and Participants

**Figure 1:**
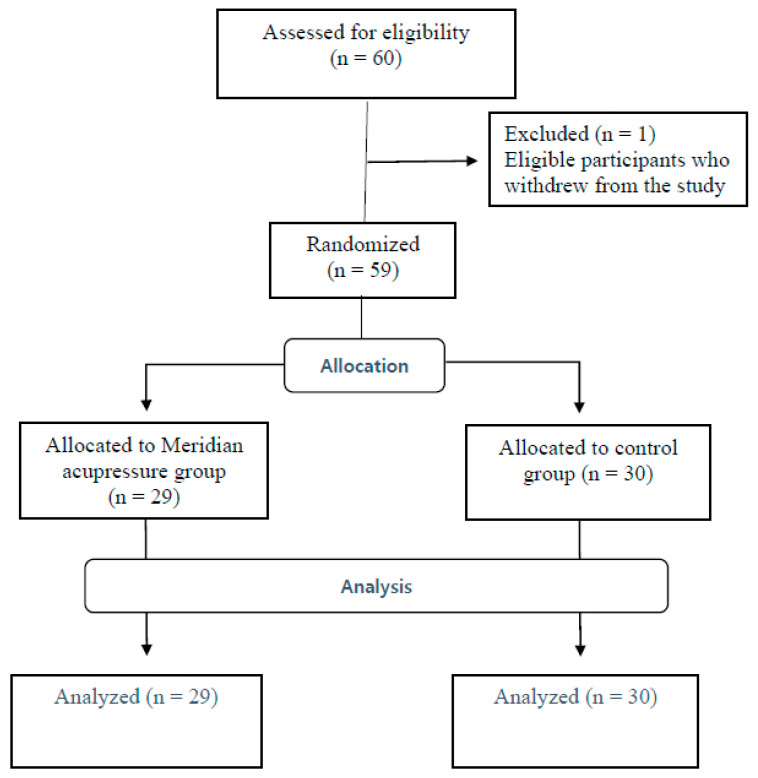
Flow diagram of subject progress.

A quasi-experimental pretest-posttest control group design was employed. The study was conducted at nurse stations in wards in S hospital located in Seoul, South Korea. Study participants were a total of 59 shiftwork nurses (intervention group: *n* = 29, control group: *n* = 30) in S hospital, Seoul. The participants were recruited through convenience sampling. During the coin toss, heads meant the subject became a participant in the intervention group in this study. The inclusion criteria for participants were daytime shiftwork nurses who voluntarily agreed to participate, without cognitive disorder, with clear consciousness, ability to communicate in verbal and non-verbal language, and ability to understand the objectives of the study. Three participants who reported a poor state of health state without a doctor’s diagnosis and prescription were included. The exclusion criteria were persons diagnosed with acute or chronic illness by a doctor, those who have taken a prescription drug within the last three months, those who have received other alternative therapies within the last month prior to the experiment, those with skin lesions at the intervention site, and pregnant and lactating women (Figure 1). Sample size adequacy (*n* = 26 in each group) using G power was estimated based on an alpha level of 0.05, effect size of 0.8, and power of 0.80 [23]. Therefore, the sample size of this study was appropriate.

### 2.2. Experimental Intervention

The six Meridian points (GV 20, GB 12, GB 21, LI 11, SI 3, KI 1) were selected to stimulate the body’s acupuncture points and Meridians, while at the same time uniting the energy and the blood through acupressure [11,12,17]. This stabilization of the harmony of blood and energy through acupressure relaxes even the small muscles deep inside the body, and activates the function of the internal organs to keep the head cool and the organs warm [11,18].

**Figure 2:**
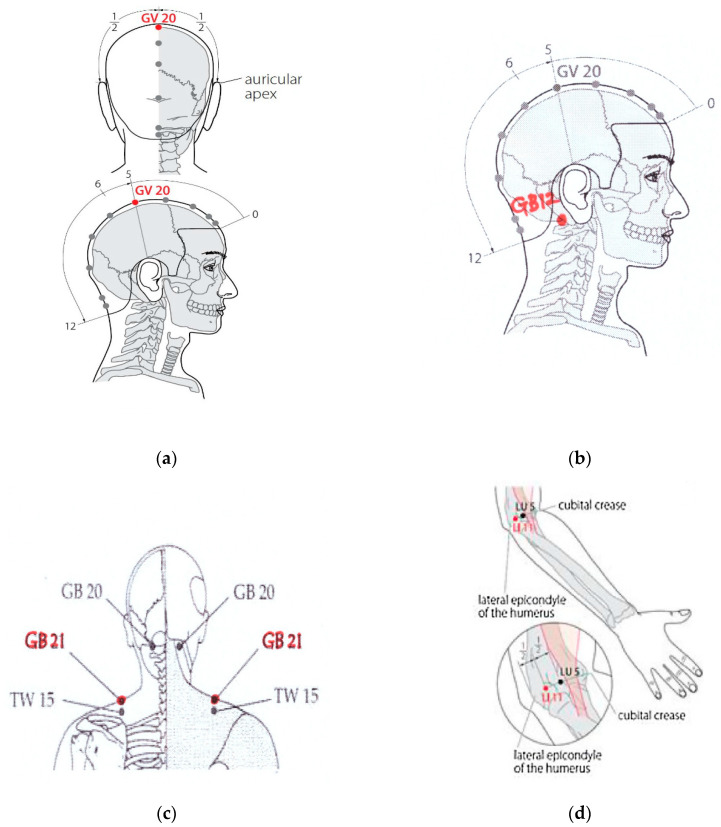

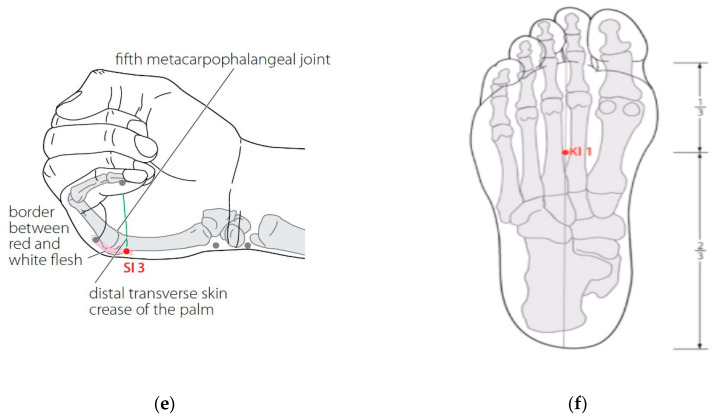
Meridian acupressure points. (**a**) GV 20 (BAIHUI); (**b**) GB 12 (WANGU); (**c**) GB 21 (JIANJING); (**d**) LI 11 (QUCHI); (**e**) SI 3 (HOUXI); (**f**) KI 1 (YONGQUAN).

Meridian acupressure as an intervention was conducted for a total of 15 min on six Meridian points (GV 20, GB 12, GB 21, LI 11, SI 3, KI 1) (Figure 2) using 2 min 30 s (10 times for 15 s at a time) on each [20,21,22]. This intervention was applied between 3~5 pm for 3 days, after the day shift, by a trained researcher.

Firstly, the researcher trimmed the nails and washed the hands immediately before the intervention in order to prevent skin irritation. The intervention was performed at the nurse station in order to maintain the privacy of the participants. A smartphone with a stopwatch function was prepared for 10-s finger-pressure per Meridian acupressure point and 5-s pause. The participants were instructed to sit comfortably on a stationary chair and were informed in advance that acupressure was to be applied on 6 Meridian points for approximately 15 min. The researcher delivering the intervention had completed a special training course as an acupressure therapist in an acupressure research institute in South Korea. This researcher has been educating and serving the community for many years. The participants were encouraged to express any discomfort during the Meridian acupressure at any time, and relaxation of mind and body was induced. The participants were allowed to take a rest for 15 min while relaxing comfortably after the intervention.

### 2.3. Measures

The study questionnaire was designed to measure the general characteristics of participants’ stress, fatigue, anxiety, and self-efficacy. General characteristics consisted of gender, age, marital status, level of education, working career, and current clinical department. This consisted of a total of 6 items.

The stress scale revised by Park and Chung [8] was validated by two nursing professors and a medical professor. The validated scale was used to measure the degree of stress of the shift nurses. It consists of a total of 30 questions using a 4 score Likert scale. This scale included two subcategories, psychological stress (15 items) and physical stress (15 items). The possible score range was 30–120, and the higher the score of the respondent, the higher the level of stress. The reliability of the scale in this study was Cronbach’s α = 0.89 (psychological stress: Cronbach’s α = 0.88, physical stress: Cronbach’s α = 0.90).

The fatigue scale was translated into a validated Korean version by Lee [24] and used to measure the degree of fatigue of participants. It consisted of a total of 17 questions using a 4 score Likert scale. This scale included three subcategories, physical fatigue (10 items), mental fatigue (2 items), and neuro-sensory fatigue (5 items). Possible score range was 17–68. The higher the score of the respondent, the higher the level of fatigue. The reliability of the scale in this study was Cronbach’s α = 0.92 (physical fatigue: Cronbach’s α = 0.92, mental fatigue: Cronbach’s α = 0.90, and neuro-sensory fatigue: Cronbach’s α = 0.94).

The State Anxiety Inventory (SAI) of the State-Trait Anxiety Inventory (STAI) developed by Spielberger [25] was translated by Kim and Shin [26] into a validated Korean SAI and used to measure the anxiety of participants. This consisted of a total of 20 questions using a 4 score Likert scale. Possible scores are 20 to 80, and the higher the score of the respondent, the higher the level of anxiety. The reliability of the scale was Cronbach’s α = 0.88.

The Self-efficacy scale developed by Sherer et al. [27] was translated into a validated Korean version by Lee [28] and used to measure the degree of self-efficacy of participants. It consisted of a total of 16 questions using a 5 score Likert scale. Possible score range was 16–80. The higher the score of the respondent, the higher the level of self-efficacy. The reliability of the scale in this study was Cronbach’s α = 0.89.

### 2.4. Procedures

Data were collected from July to August 2018. After the IRB approval, the researcher visited the department of nursing at S hospital, explained the purpose and procedure of the study, and obtained the approval for data collection. The researcher visited each ward and specialty department. The shiftwork nurses were recruited through convenience sampling, and they were randomly assigned to the intervention group or the control group. Subjects in both groups were uninformed as to which group they were assigned. The data were collected through self-report questionnaires. The pre-test included the general characteristics, stress, fatigue, anxiety, and self-efficacy level of the subjects, while the post-test included stress, fatigue, anxiety, and self-efficacy levels. After completing the questionnaire, the subjects placed it in the collection box. The Meridian acupressure provided by the researcher was given to the intervention group, while no intervention was provided to the control group. For purposes of research ethics, after completing the post-test, Meridian acupressure was given to the control group in the same manner as to the intervention group.

### 2.5. Statistical Analysis

Collected data were analyzed using the SPSS version 21.0 statistical software program (IBM, Armonk, NY, USA). The general characteristics of the participants and the homogeneity test between two groups were analyzed using descriptive statistics, independent t-test, and Chi-square test. In order to examine the effects of Meridian acupressure, mean, standard deviation, and independent t-test were used. A *p*-value of less than 0.05 was considered statistically significant.

### 2.6. Ethical Considerations

The Institutional Review Board of S hospital in Seoul, South Korea approved this study (IRB No. SEOUL 2018-06-010-002). Participants were informed that they could voluntarily take part and that they could also withdraw from participating at any time if they wished. Moreover, they were informed about the anonymity and the confidentiality of the data they would provide. The researchers obtained completed written consent forms from eligible subjects prior to their participation.

## 3. Results

### 3.1. General Characteristics of Participants and Homogeneity

Most of the participants were female, and the average age of the participants was 26.53 years old. As for marital status, the majority of the participants were single, including 27 participants (93.10%) in the intervention group and 27 participants (90.0%) in the control group. The 23 participants (79.3%) in the intervention group were university graduates, as were 20 participants (66.7%) in the control group. In terms of their career, most of the participants had 3 or more years of professional work experience (intervention: 51.8%, control: 73.4%). In terms of current clinical department, surgical medicine was the most represented in the sample (intervention: 62.1%, control: 56.7%). The analysis of the homogeneity between the intervention and control groups showed that they were homogeneous, with a significance level of *p* < 0.05 (Table 1).

### 3.2. Levels of Stress, Fatigue, Anxiety, and Self-Efficacy of Shiftwork Nurses and Homogeneity at Pre-Intervention

At pre-intervention, mean score of the participants for stress was 64.45 ± 9.13 in the intervention group and 65.80 ± 7.54 in the control group, indicating a lower level of stress in the former. Their mean score in terms of fatigue was 37.31 ± 9.74 in the intervention group and 39.43 ± 11.58 in the control group, showing a lower level of fatigue in the former. Mean score of the participants for anxiety was 49.86 ± 10.20 in the intervention group and 49.33 ± 9.24 in the control group, indicating a moderate level of anxiety. Mean score in terms of self-efficacy was 40.76 ± 10.22 in the intervention group and 41.20 ± 11.06 in the control group, showing a lower level of self-efficacy in the former. The analysis of the homogeneity of study variables between two groups showed that they were homogeneous with a significance level of *p* < 0.05 (Table 2).

### 3.3. Effects of Meridian Acupressure

The intervention group to which the Meridian acupressure was applied showed that their stress (t = 2.066, *p* = 0.043), fatigue (t = 4.590, *p <* 0.001), and anxiety (t = 2.984, *p* = 0.004) were significantly decreased compared to the control group. There was no significant difference in self-efficacy (t = 1.684, *p* = 0.098) between two groups (Table 3).

## 4. Discussion

The study was attempted in order to reduce the stress, fatigue, and anxiety of shiftwork nurses, and increase their self-efficacy by performing acupressure on six Meridian points (GV 20, GB 12, GB 21, LI 11, SI 3, KI 1) and measuring stress, fatigue, anxiety, and self-efficacy.

After dayshift work, the psychological stress score and physical stress score of the intervention group were 32.55 ± 5.56 and 31.89 ± 5.76, respectively, while the psychological stress score and physical stress score of the control group were 33.50 ± 8.59 and 32.30 ± 9.64, respectively. When considering that the psychological and physical stress scores of 20 or higher fall under the warning level [5,6,9], which is risky and requires considerable attention or evaluation from a specialist, the stress level of the subjects was very high. In the study of Choi [29], the results of the comparison between fixed shift and rotating shift nurses in regard to physical, social, sleep, and mental stress showed that the stress level of the rotating shift nurses was higher than that of the fixed shift nurses. In the present study, the psychological and physical stress scores were statistically significantly reduced as a result of performing acupressure on shiftwork nurses after their daytime shift, thereby indicating that acupressure had an effect on stress reduction. In the study of No [22] that applied four sessions of Meridian acupressure massage for two weeks (60 min per session) on ICU nurses and investigated its effects on stress and immune responses, Meridian acupressure massage reduced the subjective stress level and physiological stress response, thereby showing consistent results with those of the present study. However, the Meridian acupressure massage used in the study of No [22] took a long time, as it was applied to the whole body of the subjects for a total of 60 min (e.g., 10 min each for the abdomen, upper extremities, lower extremities, chest, face, etc.), which was difficult to perform unaided. However, the intervention of the present study is advantageous in that it reduced the stress in just 15 min, and these acupressure points can be used by the subjects themselves. Moreover, the study of Kim [30] that investigated the effects of aroma therapy hand massage, which is applied once a day for two weeks, on stress and fatigue of shiftwork nurses showed that the mean scores of stress perception and fatigue were lower than those of the control group, thereby showing a stress reduction effect in shiftwork nurses, which is consistent with the result of the present study.

The results of the acupressure performed after the daytime shift of the shiftwork nurses showed that their physical, mental, and neurosensory fatigue were statistically significantly reduced, which was the same as the result of Sim’s study [31] which investigated the positive effects of self-acupressure (GB 12, HT 7, SP 6) on sleep disorders and fatigue in clinical nurses. In the study of Lee and Lee [32], shoulder stretching and acupressure on Meridian points (GB 20, SI 9, ST 27) were applied to the outpatient nursing staff five days per week for a total of five weeks in order to examine the effects on fatigue and shoulder pain. As a result, the shoulder pain score and subjective symptoms of shoulder pain decreased, and fatigue was also reduced. In the study of Yoo [33] that applied foot reflexology massage to operating room nurses for 30 min after their shift, it was confirmed that the operating room nurses had reduced fatigue and lower extremity edema. In the study of Kang et al. [34] that confirmed the effect of Baekgaeja (white mustard seed) acupressure on the fatigue and sleep of breast cancer patients who were receiving chemotherapy, Baekgaeja (white mustard seed) was attached to Meridian points (ST 36, SP 6, HT 7), and self-acupressure was performed six times over a period of two days. As a result, fatigue decreased while sleep increased, thus showing positive effects, such as physical and psychological stability, and relaxation. As such, it can be seen that finger-pressure therapy and Meridian acupressure were effective in reducing fatigue, and they are efficient because they can be performed anywhere at no cost.

In the present study, the result of performing Meridian acupressure on shiftwork nurses after the dayshift showed that anxiety score was significantly reduced. This was similar to the results of the study of Park and Jang [11] in that holding hands and acupressure on Meridian point (PC 6) during surgery are found to be effective methods for reducing anxiety and pain. Kim and Jeong [12] studied the effects of Meridian acupressure (PC 6) on anxiety, nausea, and vomiting, as well as the physiological changes in unconscious sedated gastroscopy patients. They reported that it reduced nausea and helped maintain respiratory rate and oxygen saturation within the normal range, but it did not reduce anxiety. The study of Lee [35] which investigated the effects of Meridian acupressure (SP 6) on anxiety, pulse rate, and neonatal condition in women in childbirth reported that it was effective in reducing their level of anxiety without affecting the pulse rate and neonatal condition. The present study showed that acupressure has the effect of reducing anxiety; however, other studies reported that it is partially effective or ineffective. Therefore, Meridian acupressure needs to be applied to other subjects with anxiety or in other situations to verify its effect.

Self-efficacy, an outcome variable, slightly decreased from 40.76 ± 10.22 to 37.62 ± 10.77 as a result of acupressure after the daytime shift; however, there was no statistically significant difference. Kim [15] examined the effects of Baekgaeja (white mustard seed) applied to auricular acupressure points on the obesity index and self-efficacy in female college students by applying acupressure three times a day and maintaining Baekgaeja for four weeks. As a result, there was a reduction in body weight and body mass index, and an increase in self-efficacy. Although there is a limitation in comparison due to the small number of studies on acupressure, it is difficult to conclude that it improves self-efficacy via a short-time measurement because there are differences in the frequency and duration of Meridian acupressure.

Based on the findings of this study, Meridian acupressure can be recommended as a nursing intervention for alleviating the stress, fatigue, and anxiety of shiftwork nurses in clinical practice. Balouchi et al. [36] reported that massage-like acupressure was most often applied by nurses and a large percentage believed it useful for treating illness. Meridian Acupressure may be done by the nurses themselves for self-health care. Meridian acupressure can be performed without the limitation of time or place, although a systematic nursing intervention program using Meridian acupressure that can be used in local communities should be developed.

In the future, replication studies using Meridian acupressure as an intervention need to be conducted with an increased number of samples by selecting a greater number of hospitals, targeting various work environments and subjects, and including a variety of study parameters. It is necessary to identify the anxiety status of subjects more accurately by measuring the subject’s anxiety traits in future research. Furthermore, experimental studies also need to be performed on self-efficacy, which is an important factor for nurses, by increasing the frequency and duration of the Meridian acupressure.

There are some study limitations. It is difficult to generalize the results of this study since the study population was limited to shiftwork nurses, and working environment and workload are different for each hospital. In addition, the trait anxiety score could not be completely controlled because it was not measured. In addition, there was no active control group such as “sham acupressure”, which would have contributed to a much stronger experimental design, or at least a “Time and Attention” control where the study participants in the control group read about acupuncture points and the effects they have on the body in terms of stress, fatigue, anxiety, and self-efficacy. However, the main purpose of this study is to provide information for further research.

## 5. Conclusions

In this study, Meridian acupressure was applied to shiftwork nurses, and was reported to be effective in reducing stress, fatigue, and anxiety. Therefore, it is necessary to apply Meridian acupressure to nurses in various clinical settings. The present study is significant in that Meridian acupressure could be appropriately used as a nursing intervention method to reduce stress, fatigue, and anxiety of shiftwork nurses.

## Figures and Tables

**Table 1:** General characteristics of participants and homogeneity.

Characteristics	Intervention Group (N = 29) *n* (%)	Control Group (N = 30) *n* (%)	x^2^/t	*p*
Gender			1.147 ^†^	0.353
Female	26 (89.7)	29 (96.7)		
Male	3 (10.3)	1 (3.3)		
Age (year)			0.023 ^†^ −0.844	0.993 0.402
20–25	11 (37.9)	11 (36.7)		
26–30	16 (55.2)	17 (56.7)		
≥31	2 (6.9)	2 (6.6)		
Mean ± SD in each group	26.24 ± 2.39	26.80 ± 2.68		
Mean ± SD in total	26.53 ± 2.54			
Marital status			0.183 ^†^	1.000
Single	27 (93.1)	27 (90.0)		
Married	2 (6.9)	3 (10.0)		
Child(ren)			1.052 ^†^	0.492
Yes	1 (3.4)	0 (0.0)		
No	28 (96.6)	30 (100.0)		
Level of education			1.193	0.382
College	6 (20.7)	10 (33.3)		
University	23 (79.3)	20 (66.7)		
Working career (year)			4.347 ^†^	0.226
<1	9 (31.0)	3 (10.0)		
1–<3	5 (17.2)	5 (16.6)		
3–<5	7 (24.2)	11 (36.7)		
≥5	8 (27.6)	11 (36.7)		
Current clinical department			0.178	0.792
Gastroenterology	11 (37.9)	13 (43.3)		
Surgical medicine	18 (62.1)	17 (56.7)		
Health state			0.408 ^†^	0.816
Good	20 (69.0)	21 (70.0)		
Moderate	7 (24.1)	8 (26.7)		
Poor	2 (6.9)	1 (3.3)		

^†^ Fisher’s exact test.

**Table 2:** Levels of stress, fatigue, anxiety, and self-efficacy of shiftwork nurses, and homogeneity at pre-intervention.

Variables		Intervention Group (N = 29) Mean ± SD	Control Group (N = 30) Mean ± SD	t	*p*
Stress	Psychological	32.55 ± 5.56	33.50 ± 8.59	−0.502	0.618
	Physical	31.89 ± 5.76	32.30 ± 9.64	−0.194	0.847
	Total	64.45 ± 9.13	65.80 ± 7.54	−0.369	0.713
Fatigue	Physical	23.07 ± 5.93	24.10 ± 6.45	−0.638	0.526
	Mental	4.59 ± 1.57	5.00 ± 1.78	−0.945	0.348
	Neuro-sensory	9.66 ± 2.97	10.33 ± 3.91	−0.747	0.458
	Total	37.31 ± 9.74	39.43 ± 11.58	−0.761	0.450
Anxiety		49.86 ± 10.20	49.33 ± 9.24	0.209	0.835
Self-efficacy		40.76 ± 10.22	41.20 ± 11.06	−0.159	0.874

**Table 3:** Effects of Meridian acupressure.

Variables			Pre Test Mean ± SD	Post Test Mean ± SD	Mean Difference Mean ± SD	t	*p*
Stress	Psychological	Inter. N = 29	32.55 ± 5.56	23.51 ± 5.70	9.03 ± 6.05	2.038	0.046 *
		Cont. N = 30	33.50 ± 8.59	28.56 ± 9.39	4.93 ± 9.05		
	Physical	Inter. N = 29	31.89 ± 5.76	24.34 ± 5.70	8.96 ± 5.77	2.437	0.018 *
		Cont. N = 30	32.30 ± 9.64	27.23 ± 7.21	5.06 ± 6.48		
	Total	Inter. N = 29	64.45 ± 9.13	47.86 ± 10.39	16.58 ± 10.24	2.066	0.043 *
		Cont. N = 30	65.80 ± 7.54	55.80 ± 15.62	10.00 ± 13.89		
Fatigue	Physical	Inter. N = 29	23.07 ± 5.93	16.75 ± 4.29	6.31 ± 4.00	5.222	<0.001
		Cont. N = 30	24.10 ± 6.45	22.83 ± 5.93	1.26 ± 3.40		
	Mental	Inter. N = 29	4.59 ± 1.57	2.68 ± 1.10	1.91 ± 1.26	4.822	<0.001
		Cont. N = 30	5.00 ± 1.78	4.43 ± 1.63	0.57 ± 2.13		
	Neuro-sensory	Inter. N = 29	9.66 ± 2.97	8.17 ± 3.08	1.48 ± 1.68	3.445	0.001
		Cont. N = 30	10.33 ± 3.91	10.70 ± 3.12	−0.36 ± 2.37		
	Total	Inter. N = 29	37.31 ± 9.74	28.62 ± 7.65	8.68 ± 5.68	4.590	<0.001
		Cont. N = 30	39.43 ± 11.58	37.96 ± 9.94	1.46 ± 6.36		
Anxiety		Inter. N = 29	49.86 ± 10.20	41.13 ± 8.95	8.72 ± 7.55	2.984	0.004 *
		Cont. N = 30	49.33 ± 9.24	46.10 ± 9.90	3.23 ± 6.55		
Self-efficacy		Inter. N = 29	40.76 ± 10.22	37.62 ± 10.77	3.13 ± 6.00	1.684	0.098
		Cont. N = 30	41.20 ± 11.06	42.53 ± 12.19	−1.33 ± 13.02		

Inter. = Intervention group; Cont. = Control group; * *p* < 0.05.

## Data Availability

No new data were created or analyzed in this study. Data sharing is not applicable to this article.
